# Supplementary material for: Safety and Efficacy of Hypofractionated Stereotactic Radiotherapy with Anlotinib Targeted Therapy for Glioblastoma at the First Recurrence: A Preliminary Report
Source: Brain Sci. 2022 Apr 2;12(4):471. doi: 10.3390/brainsci12040471 (PMC9032064; doi:10.3390/brainsci12040471)
Supplement: Supplementary file 1 [file brainsci-12-00471-s001.zip › brainsci-1573531-supplementary.pdf]

Supplementary Materials:

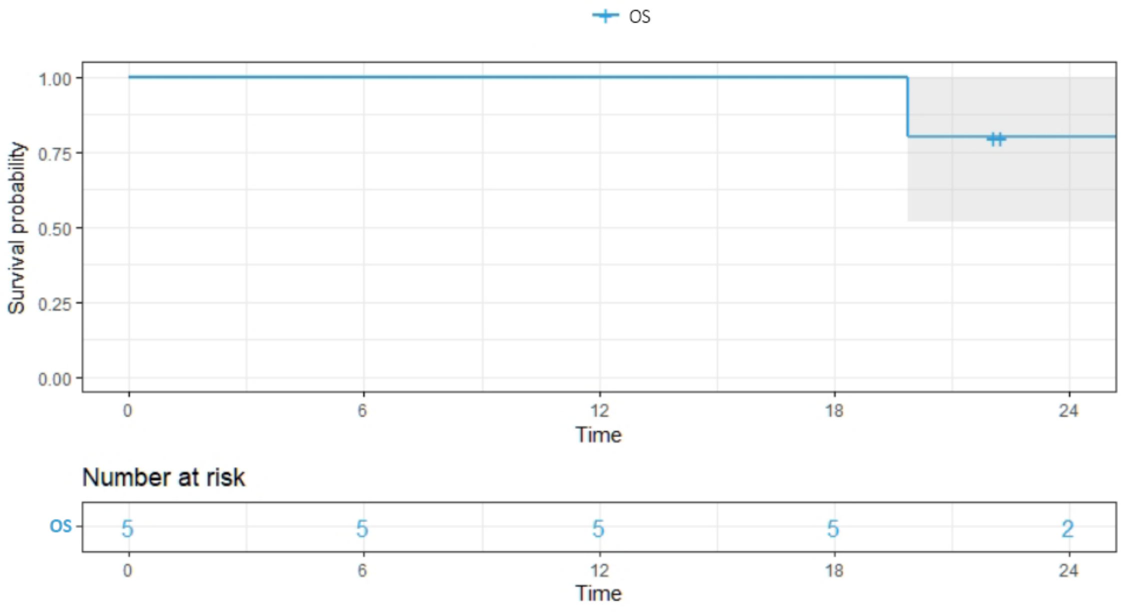

Figure S1: Overall survival (OS) from salvage treatment of all rHGG patients (calculated with the Kaplan–Meier method).

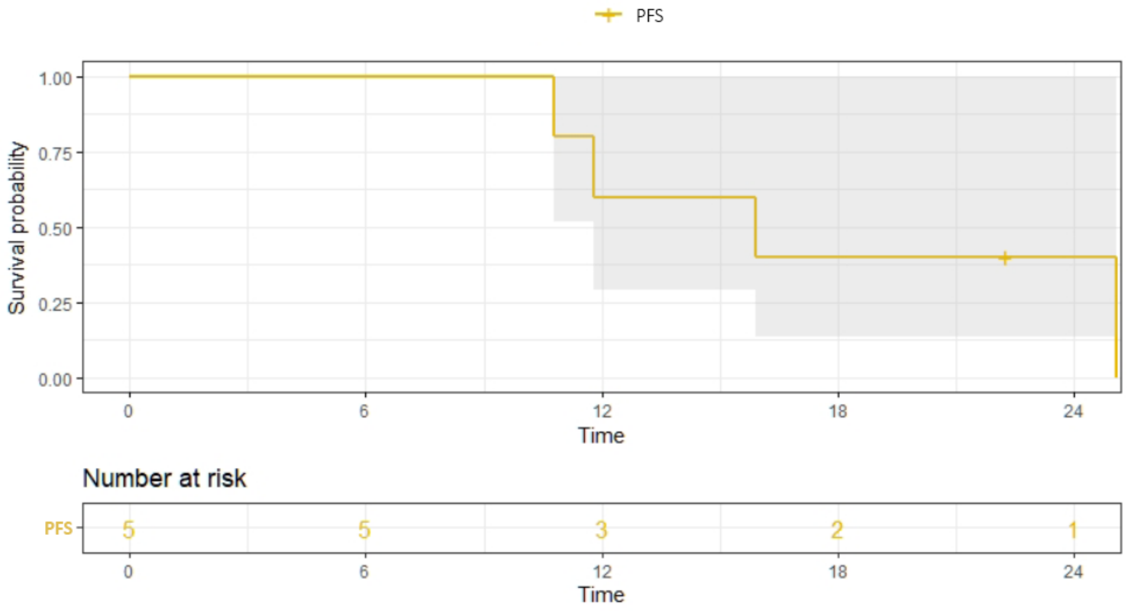

Figure S2: Progression-free survival (PFS) from salvage treatment of all rHGG patients (calculated with the Kaplan–Meier method).
